# Analysis of Longitudinal Change Patterns in Developing Brain Using Functional and Structural Magnetic Resonance Imaging via Multimodal Fusion

**DOI:** 10.1002/hbm.70241

**Published:** 2025-07-03

**Authors:** Rekha Saha, Debbrata K. Saha, Zening Fu, Marlena Duda, Rogers F. Silva, Tony W. Wilson, Yu‐Ping Wang, Julia M. Stephen, Vince D. Calhoun

**Affiliations:** ^1^ Tri‐Institutional Center for Translational Research in Neuroimaging and Data Science (TReNDS) Georgia State University, Georgia Institute of Technology, and Emory University Atlanta Georgia USA; ^2^ Institute for Human Neuroscience, Boys Town National Research Hospital Omaha Nebraska USA; ^3^ Department of Biomedical Engineering Tulane University New Orleans Louisiana USA; ^4^ Mind Research Network Albuquerque New Mexico USA

## Abstract

Functional and structural magnetic resonance imaging (fMRI and sMRI) are complementary approaches that can be used to study longitudinal brain changes in adolescents. Each individual modality offers distinct insights into the brain. However each individual modality may overlook crucial aspects of brain analysis. By combining them, we can uncover hidden brain connections and gain a more comprehensive understanding. In previous work, we identified multivariate patterns of change in whole‐brain function during adolescence. In this work, we focus on linking functional change patterns (FCPs) to brain structure. We introduced two approaches and applied them to data from the adolescent brain and cognitive development (ABCD) dataset. First, we evaluate voxel‐wise sMRI‐${\mathrm{FCP}}_{\text{asym}}$ coupling to identify structural patterns linked to our previously identified FCPs. Our approach revealed multiple interesting patterns in functional network connectivity (FNC) and gray matter volume (GMV) data that were linked to subject‐level variation. ${\mathrm{FCP}}_{\text{asym}}$ components 2 and 4 exhibit extensive associations between their loadings and voxel‐wise GMV data. Secondly, we leveraged a symmetric multimodal fusion technique called multiset canonical correlation analysis (mCCA) + joint independent component analysis (jICA). Using this approach, we identified structured ${\text{FCPs}}_{\mathrm{sym}}$ such as one showing increased connectivity between visual and sensorimotor domains and decreased connectivity between sensorimotor and cognitive control domains, linked to structural change patterns (${\text{SCPs}}_{\mathrm{sym}}$) including alterations in the bilateral sensorimotor cortex. Interestingly, females show stronger connection between brain functional and structural changes than males, highlighting gender‐related differences. The combined results from both asymmetric and symmetric multimodal fusion methods underscore the intricate gender‐specific nuances in neural dynamics. By utilizing two complementary multimodal approaches, our study enhances our understanding of the evolving nature of whole brain connectivity and structure during the adolescent period, shedding light on the nuanced processes underlying adolescent brain development.

## Graphical abstract

In this study, we propose both asymmetric and symmetric multimodal fusion techniques to investigate longitudinal brain change patterns in adolescents. In our analysis, multivariate patterns of functional network connectivity and gray matter volume (GVM) demonstrate relationships with increased age. In asymmetric multimodal fusion, two functional change patterns (FCP) components exhibit extensive associations between their loadings and voxel‐wise GMV data. In symmetric multimodal fusion, one FCP component shows increased connectivity between visual and sensorimotor domains and decreased connectivity between sensorimotor and cognitive control domains, linked to structural change patterns including alterations in the bilateral sensorimotor cortex.

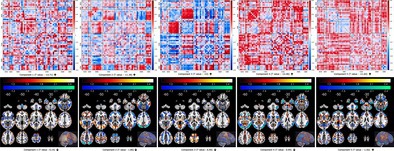

## 1 Introduction

The intricate neural network within the brain is recognized as one of the most complex systems in existence. A modern neuroscience approach involves viewing brain regional interactions as a network, known as the brain connectome or brain network (Kong and Yu 2014). By understanding network properties, researchers can gain insight into how the brain manages information flow by transferring neural signals among brain regions (Mesulam 2000). Magnetic resonance imaging (MRI) is a tremendously used method for obtaining comprehensive brain information and one of the only modalities that is capable of visualizing both structure and function. Structural neuroimaging modalities, such as sMRI and diffusion MRI, offer insights into the brain's anatomical structure and tissue composition, while functional neuroimaging modalities, such as fMRI based on the blood‐oxygenation‐level‐dependent signal, provide indirect measurements of brain function and activity (Abrol et al. 2017; Ogawa et al. 1990). fMRI is a key method for assessing functional connectivity (FC) by analyzing temporal coherence among different brain regions using resting‐state data. Most studies have still examined functional and structural measures separately when analyzing the brain. However, there is considerable evidence that combining structural and functional MRI data can lead to benefits (Calhoun and Adali 2008; Rykhlevskaia et al. 2008). Multimodal fusion in neuroimaging integrates information obtained from various imaging techniques, aiming to overcome individual modality limitations and gain deeper insights into brain structure and function (Calhoun and Sui 2016; Sui et al. 2014; Zhang et al. 2011). The primary goal of multimodal fusion is to enhance the analytical capabilities of each modality through combined analysis, rather than treating each modality separately.

Prior research in multimodal analysis often involves examining data from distinct modalities separately and later combining the independent outcomes obtained from these individual analyses. Alternatively, some studies have used information from one modality to constrain or guide models related to another modality. While these approaches have been beneficial, they can underutilize cross‐modality information. There is a growing trend in approaches that employ symmetric data fusion methods (Calhoun and Sui 2016) to capitalize on joint information across modalities. Feature‐based symmetric data fusion methods initially extract valuable, high‐dimensional features from diverse modalities and then explore the connections among these features. This method effectively uses the complementary information present in each modality to reveal variations in data that might not be evident through unimodal analyses. Various studies have highlighted the potential of employing such cross‐modality or joint information in understanding the human brain and its disorders. These methods have been instrumental in characterizing diseases, identifying potential biomarkers, and unraveling disrupted connections in complex mental illnesses (Calhoun and Sui 2016).

Each multivariate fusion method possesses distinct optimization priorities and limitations. For instance, methods like mCCA (Correa et al. 2009) and partial least squares (PLS) (Chen et al. 2009; Lin et al. 2003) facilitate both common and distinct levels of connection across modalities. However, these methods may not achieve adequate spatial sparsity in their separated sources. For instance, mCCA emphasizes intersubject covariation across two feature sets, generating linked variables known as canonical variants (CVs). These CVs correlate with each other solely on the same indices, and their corresponding correlation values are termed canonical correlation coefficients (CCC). While this approach captures both common and distinct aspects of features, the resulting brain maps for multiple components may appear similar if the CCCs lack sufficient distinctiveness. On the other hand, spatial decomposition approaches like jICA (Calhoun et al. 2006) and linked independent component analysis (Groves et al. 2011) maximize independence among estimated sources combining multiple modalities, but they primarily allow a common mixing matrix. While these methods detect common features across all modalities effectively, they may neglect features distinct to one or more modalities, especially when combining more than two modalities. Several prior studies that merged function and structure (Camara et al. 2010; Olesen et al. 2003; Rykhlevskaia et al. 2008; Sui et al. 2012) support the idea that components derived from each modality exhibit some correlation in their mixing profiles among subjects. This serves as motivation to utilize an approach that aims for optimal intermodal association flexibility while ensuring robust source separation capabilities.

To analyze shared information among features from different imaging modalities, we employed the multiset canonical correlation analysis + joint independent component analysis (mCCA + jICA) method (Sui et al. 2011). This widely recognized and extensively used data‐driven multivariate multimodal fusion approach (Calhoun et al. 2006; Correa et al. 2010) integrates mCCA and jICA in a two‐step process (Calhoun and Sui 2016). Initially, mCCA identifies highly correlated components across multiple modalities (Sui et al. 2011; Sui et al. 2013). Subsequently, jICA decomposes these correlated components into spatially independent components (ICs). The mCCA + jICA algorithm has been utilized (Sui et al. 2011) to combine fMRI contrast maps and diffusion tensor imaging (DTI) fractional anisotropy (FA) maps, enhancing the accuracy of group classification among healthy controls (HCs), schizophrenia patients (SPs), and bipolar patients (BPs) compared to using the algorithms individually. Previous applications include Ouyang et al., who identified patterns of gray matter (GM) and white matter (WM) covariance in Alzheimer's disease patients (Ouyang et al. 2015). Similarly, Kim et al. applied mCCA + jICA to analyze multimodal sMRI and DTI data from HCs and patients with obsessive–compulsive disorder, uncovering significant alterations in interconnected networks of GM and WM (Kim et al. 2015). But, to the best of our knowledge, no prior studies have investigated the estimation of changes in multivariate pattern coupling in FNC and GMV associated with age progression, utilizing the mCCA + jICA multimodal fusion analysis method.

In our study, we present two innovative methodologies: (1) explore the relationship between multivariate functional change patterns (${\text{FCPs}}_{\text{asym}}$) and voxel‐wise gray matter volume (ΔGMV) data to investigate age‐related changes in whole‐brain structure and function within individuals (2) employ a symmetric multimodal fusion technique mCCA + jICA to uncover structural change patterns (${\text{SCPs}}_{\mathrm{sym}}$) associated with ${\text{FCPs}}_{\mathrm{sym}}$ using FNC matrices and GMV data from the ABCD dataset, comprising over 11,000 adolescent subjects across multiple scans. For each subject, we compute cell‐wise ΔFNC and ΔGMV matrices, followed by estimating covarying multivariate patterns. Without predefined restrictions to specific seed regions, we calculate voxel‐wise correlations between functional data loading parameters and ΔGMV data. The second approach involves using the mCCA + jICA multimodal fusion technique to estimate covarying multivariate patterns between ${\text{FCPs}}_{\mathrm{sym}}$ and ${\text{SCPs}}_{\mathrm{sym}}$.

Through both symmetric and asymmetric multimodal fusion techniques, our analysis identifies FNC linked to GMV data, suggesting concurrent changes between functional connectivity and structural data during adolescence. Notably, gender‐related differences reveal stronger coupling between brain functional and structural changes in females compared to males. Our statistical analysis unveils several FCPs, and SCPs associated with longitudinal changes in psychopathology and cognition scores within the developing brain. The remainder of the research paper is organized as follows: in the Materials and Methods section, we present the data preprocessing and analysis procedures. In the Results section, we showcase changes in brain functional and structural coupling with age and the connections between various FCPs and SCPs with gender, differences in psychopathology, and cognition scores. Finally, in the Discussion and Conclusion section, we delve into the implications of our findings.

## 2 Materials and Methods

### 2.1 ABCD Data Summary

The study utilizes data collected by the ABCD study^1^. The primary objective of the ABCD study is to monitor brain development during adolescence. To realize this, the study collected a diverse array of data to discern the influences of biological and environmental factors on developmental trajectories. The ABCD study is the largest long‐term investigation of brain development and child health in the United States. It includes multisession MRI scans from over 11,800 children aged 9–11 years at baseline. Table 1 provides the demographic information for the ABCD dataset. The dataset covers subject details such as social, emotional, and cognitive development, gender identity, physical and mental health assessments, and medical backgrounds. Ethical considerations were upheld through parental informed consent and child assent, all approved by the Institutional Review Board (IRB). The ABCD dataset is accessible via the National Institute of Mental Health Data Archive (NDA)^2^, which has been made available as an open‐source resource following its compilation from a diverse range of research endeavors across various scientific domains. Collaborating with major MRI system manufacturers (Siemens, General Electric, and Philips), the data were collected from 21 sites across the United States, ensuring standardized imaging methods. The TR was 800 ms, with a resolution of 2.4 × 2.4 × 2.4 mm. More information about imaging parameters can be found at^3^. The quality of data was maintained through standard fMRI preprocessing and the NeuroMark framework (Du et al. 2020), a fully automated independent component analysis (ICA)‐based approach that identifies brain networks across subjects. The present study utilized data from 2734 subjects who have both baseline and 2‐year follow‐up scanned data of both the FNC and gray matter volume data. In our analysis, we specifically focused on the first scan from both the baseline and 2‐year follow‐up data.

**TABLE 1:** ABCD data demographic information.

Event name	Subject	Gender (F/M)	Age (month)	Weight (lb)	Height (inch)	Race (W/H/B/O/A)
Baseline	11,244	5347/5833	119 ± 8	82.5 ± 23.7	55.3 ± 3.3	4771/1863/1325/929/181
Second year	3678	1589/1875	143 ± 8	106.6 ± 31.4	60.1 ± 3.8	1631/629/317/284/57

Abbreviations: A, Asian; b, black; F, female; H, Hispanic; M, male; O, others/unknown; W, white.

### 2.2 Image Preprocessing of FMRI Data

We conducted preprocessing on the FastTrack fMRI images using a combination of the FMRIB Software Library v6.0 (FSL) toolbox and Statistical Parametric Mapping 12 (SPM) toolbox within the MATLAB 2019b environment. Initially, we corrected for rigid body motion by employing the mcflirt tool in FSL. Then, we conducted distortion correction utilizing fMRI field map data that were collected with phase‐reversed blips. This process generated pairs of images with distortion occurring in opposing directions. To estimate the susceptibility‐induced off‐resonance field, we employed the FSL tool topup, utilizing volumes acquired with phase encoding both in the anterior–posterior and posterior–anterior directions. The coefficients derived from the output field map were then applied to correct distortion in the fMRI volume using the FSL tool applytopup. In the following step, we removed the initial 10 scans with substantial signal changes to allow the tissue to stabilize in terms of radio frequency excitation. Subsequently, we spatially aligned the fMRI data to the standard Montreal Neurological Institute (MNI) space, utilizing the echo‐planar imaging template and resampling the data to 3 × 3 × 3 mm isotropic voxels via the spatial normalization tool in SPM. Lastly, we applied Gaussian smoothing with a full width at half maximum (FWHM) of 6 mm to the resliced fMRI images.

### 2.3 Quality Control (QC)

We conducted data quality control (QC) on the preprocessed fMRI images to select subject data for subsequent analysis. The quality of how well subjects' data were normalized to the MNI space has a significant impact on both the results of ICA and the estimation of FNC. Consequently, we excluded scans that did not exhibit satisfactory normalization to the MNI standard space. To be more specific, we compared individual masks with a group mask and retained scans that demonstrated strong similarities between their individual masks and the group mask. To achieve this, we initially calculated an individual mask for each scan of each subject based on the first fMRI time volume. Voxels were set to 1 if they exceeded 90% of the mean signal across the entire brain. Subsequently, we generated a group mask by designating voxels as 1 if they had more than 90% agreement with individual masks across the scans. For each scan, we computed spatial correlations between the group mask and the individual mask, focusing on the top 10 slices, the bottom 10 slices, and the entire mask. This resulted in three correlation values for each scan. We included scans for further analysis if they met the following criteria: top‐10‐slices correlation greater than 0.75, bottom‐10‐slices correlation exceeding 0.55, and whole‐brain correlation surpassing 0.8. This method ensures the inclusion of high‐quality masks and fMRI data for retained scans, building on its success in previous research.

### 2.4 Neuromark Framework

To capture reliable intrinsic connectivity networks (ICNs) and their corresponding time courses (TCs) across each subject, we utilized a robust and fully automated independent component analysis (ICA)‐based framework known as NeuroMarkfMRI_—_1.0 network templates was applied to the ABCD data. The NeuroMark_—_fMRI_—_1.0 network templates were constructed based on two healthy control datasets: the Human Connectome Project (HCP, 823 subjects after selection) and the Genomics Superstruct Project (GSP, 1005 subjects after selection). Further details about the NeuroMark framework and templates are available in the GIFT toolbox^4^ and at^5^ (Du et al. 2020). The selected spatial priors have been shown to be highly reliable across different pipelines and various adult and adolescent datasets and populations (DeRamus et al. 2021). This approach yields 53 ICNs for each subject, with the resulting networks being highly consistent and comparable across subjects, sessions, and scans. Children's data can be noisy with more confounding effects, such as larger head motions. To mitigate this, we included four additional postprocessing steps to carefully regress out the remaining noise in the time courses (TCs) of the ICNs: (1) detrending linear, quadratic, and cubic trends, (2) removing detected outliers, (3) performing multiple regression to remove variance linked to head motion parameters (three rotations and three translations) and their derivatives, and (4) applying bandpass filtering with a cutoff frequency of 0.01–0.15 Hz. After the postprocessing, we calculated Pearson correlation coefficients between postprocessed TCs to estimate the static FNC for each scan.

### 2.5 Preprocessing of SMRI Data

We conducted preprocessing on the sMRI data using statistical parametric mapping^6^ within the MATLAB 2020b environment. The structural images were subjected to segmentation into gray matter, white matter, and CSF with additional modulation by the Jacobian to produce voxel‐wise gray matter volume (GMV) maps. Subsequently, the GMV maps underwent smoothing via a Gaussian kernel with a FWHM of 6 mm.

### 2.6 Analysis of Longitudinal Change Patterns in FNC and GMV

In our research, we utilized subject‐specific fMRI and sMRI data acquired during both the baseline and 2‐year follow‐up scans to investigate changes in FNC and GMV. We calculated the cell‐wise differences between the baseline and 2‐year follow‐up FNC and GMV data to create ΔFNC and ΔGMV matrices, respectively, representing the changes in FNC and GMV over time. These matrices were then analyzed using both asymmetric and symmetric fusion approaches to find link between brain functional connectivity and structure. In the asymmetric fusion approach, we applied the ICA using the infomax algorithm (Bell and Sejnowski 1995) to deconstruct the ΔFNC in order to recognize longitudinal brain functional coupling and capture covarying patterns of changes, which are called ${\mathrm{FCP}}_{\text{asym}}$. We extended our analysis to investigate the connection between ${\mathrm{FCP}}_{\text{asym}}$ and brain structure. This involved calculating the voxel‐wise correlation between the raw ΔGMV data and the loading parameters obtained from functional data after ICA estimation. More precisely, the second‐level ICA model equation can be expressed as:

(1) X=A⋅S

The following effectively represents the functional input data for the ICA model as:

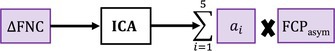

Next, we applied a symmetric fusion approach via mCCA + jICA to estimate joint ${\text{SCPs}}_{\mathrm{sym}}$ and ${\text{FCPs}}_{\mathrm{sym}}$. In the symmetric fusion approach, we run mCCA + jICA method to deconstruct the ΔFNC and ΔGMV matrices and identify patterns of change, namely, functional and structural change patterns for FNC and GMV, respectively. We determined the optimal number of components, selecting five components for both GMV and FNC data using the elbow criteria. The mCCA + jICA model equation utilized in our experiment is expressed as follows:

(2) $$X_{k}=A_{k}\cdot S_{k}$$

In this analysis, the data matrix *X* has dimensions of 2734 (subjects) × cells (representing either the upper triangular elements of the ΔFNC matrix for fMRI or the number of voxels for sMRI). The matrix *A* has dimensions of 2734 × 5 (components), *S* is 5 × cells (components), and the mCCA + jICA model involves *k* = 2 modalities.

This effectively represents the input data for the mCCA + jICA approach as:

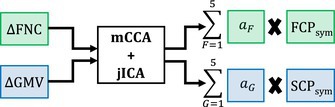

**FIGURE 1:**
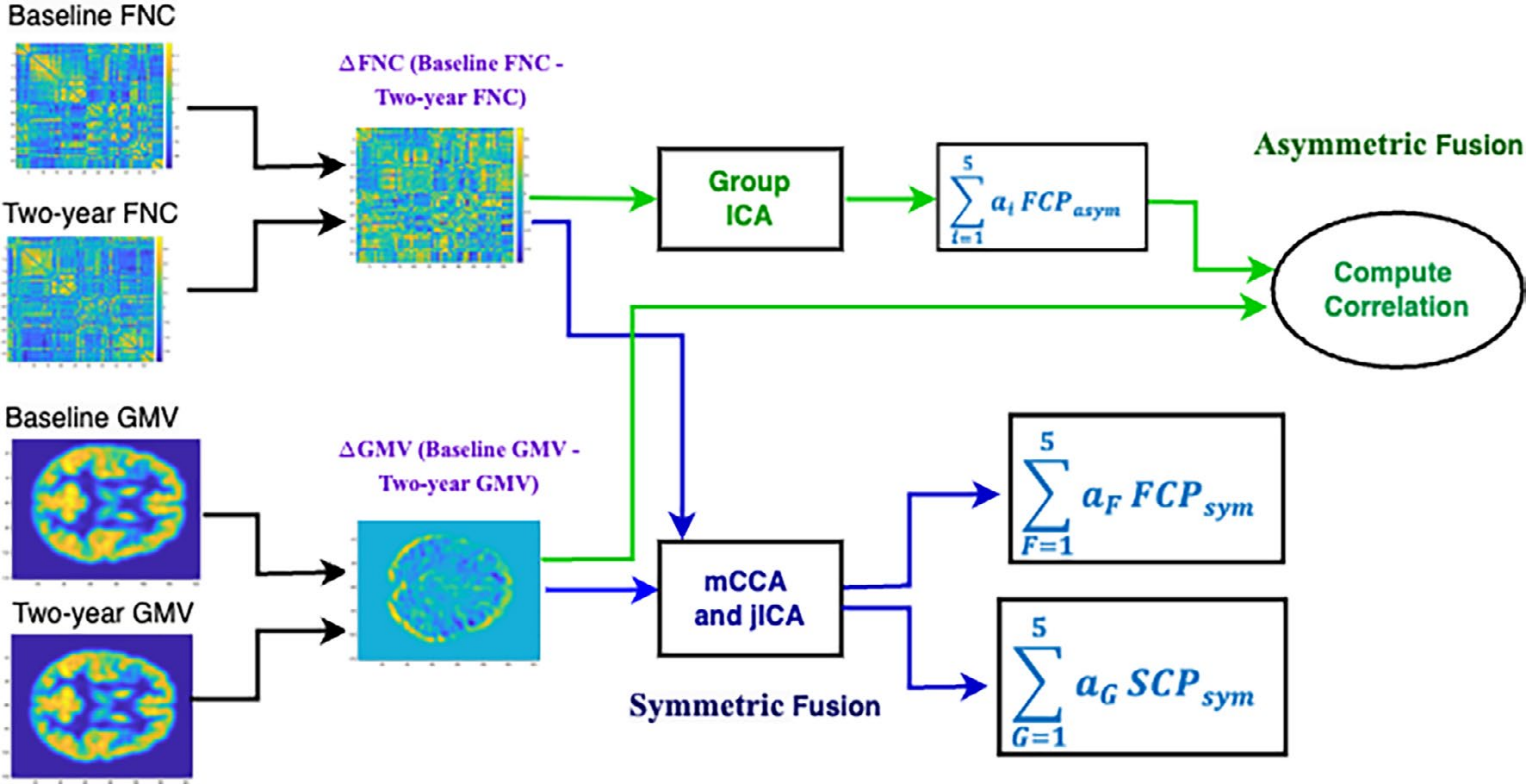
Block diagram of longitudinal multimodal functional and structural change pattern coupling analysis workflow. The subject‐specific ΔFNC and ΔGMV matrices were constructed by calculating the difference between preprocessed baseline and 2‐year FNC and GMV data.

Here, ΔFNC denotes the difference between baseline ($F_{0}$) and 2‐year follow‐up ($F_{2}$) functional network connectivity data, while ΔGMV corresponds to the difference between baseline ($G_{0}$) and 2‐year follow‐up ($G_{2}$) gray matter data. The source matrices of FCPs and SCPs capture maximally independent patterns of functional and structural changes, respectively. The parameter $a_{i}$ is each component's subject‐specific loading parameters of FNC data in the ICA model. The terms $a_{F}$ and $a_{G}$ denote the subject‐specific loading parameters for each component in the FNC and GMV data, respectively, in the context of the mCCA + jICA approach. These loading parameters quantify the individual subject's contribution to the respective components. A block diagram of the analysis workflow is shown in Figure 1. Moreover, following the mCCA + jICA estimation, we proceeded to evaluate the loading parameters and source matrix. In order to identify ${\text{FCPs}}_{\mathrm{sym}}$ and ${\text{SCPs}}_{\mathrm{sym}}$ that showed significant longitudinal changes compared to zero, we conducted one‐sample *t*‐tests on the loading parameters $a_{F}$ and $a_{G}$ for both modalities. We also conducted a one‐sample *t*‐test on the loading parameters ($a_{i}$) following the ICA estimation. Statistical significance was assessed at a 95% confidence level, with adjustments made for multiple comparisons using a false discovery rate (FDR) approach.

### 2.7 Gender‐Based Multimodal Fusion Analysis

For the asymmetric multimodal fusion approach, we calculated correlations between ΔGMV data and loading parameters of functional data separately for males and females to examine gender differences in coupling. The next step involved computing the difference between these correlations to understand the gender effect. In the symmetric multimodal fusion approach, we segregated the male and female loadings for both GMV and FNC data. Subsequently, we computed correlations between GMV male loadings and FNC male loadings, and between GMV female loadings and FNC female loadings. By quantifying the difference between these correlations (female—male), we assessed the strength of coupling between GMV and FNC loadings relative to gender. In both approaches, a significant positive difference would indicate a stronger coupling between functional and structural data in females compared to males.

### 2.8 Quartile Analysis of Pattern Changes via Relationship to Subject Measures

In order to reduce the number of comparisons, we calculated composite scores for both cognitive and psychopathology assessments. We used the same variables and procedures to compute the composite cognitive and psychopathology assessments as in our previous work (See supplementary material for details) (Fu et al. n.d.; Saha et al. 2024). Specifically, we computed the subject‐wise differences between baseline and 2‐year data for composite psychopathology and cognitive scores, representing changes in psychopathology and cognitive performance, respectively. To explore the relationship among subjects who displayed the most significant age‐related change pattern association with structural data, we conducted an analysis of the connection between loading parameters of ICA and ΔGMV data. We specifically selected subjects whose changes in psychopathology scores fell within or below the lower quartile. We then computed the correlation between the loading parameters of ${\text{FCPs}}_{\text{asym}}$ and the raw ΔGMV data for these chosen subjects, creating a voxel‐wise association map for the lower quartile. Following the same procedure for subjects in the upper quartile, we also generated an upper quartile voxel‐wise association map. Finally, we calculated the disparity between the upper and lower quartile voxel‐wise correlation maps. The same methodology was applied to evaluate differences in cognitive scores. It is important to note that all our findings underwent correction for multiple comparisons using the FDR (Benjamini and Hochberg 1995).

## 3 Results

### 3.1 Structure–Function Coupling Strengthens With Age Across Development

**FIGURE 2:**
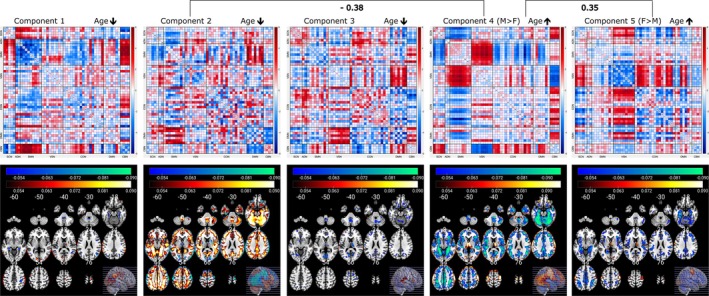
FNC component plots are displayed on the top, along with spatial maps depicting voxel‐wise GMV correlation with the loading parameters of the ${\text{FCPs}}_{\text{asym}}$ are shown at the bottom for each component (asymmetric fusion). In the figure, we observe the voxel‐wise correlation for Components 2 and 4 have the highest positive (Component 2) and negative (Component 4) values.

**FIGURE 3:**
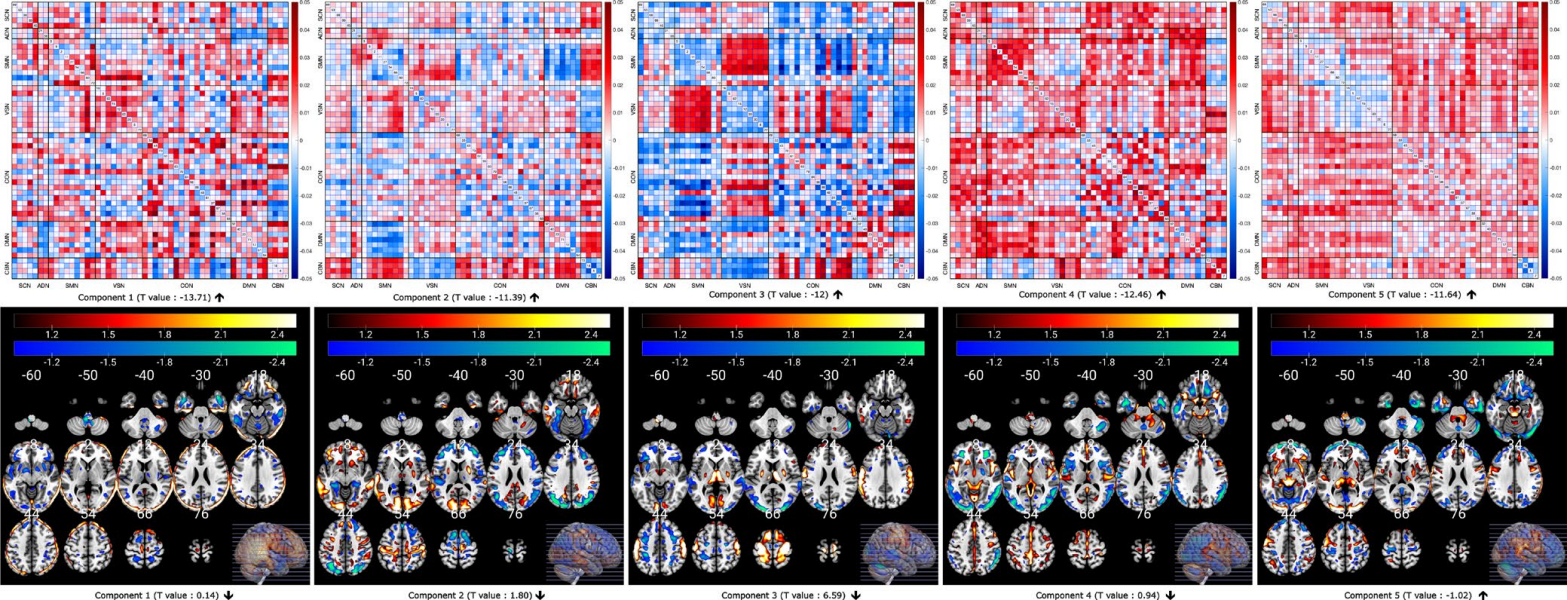
FNC components and spatial map of GMV components for the mCCA + jICA symmetric fusion approach. Component 3 from both the functional and structural data showing highly structured change patterns. In the figure, we observe increased functional connectivity between VS and SM domains or decreased functional connectivity between SM and CC correlated with ${\text{SCPs}}_{\mathrm{sym}}$ indicating alterations in the bilateral sensorimotor cortex.

The NeuroMark_—_fMRI_—_1.0 template encompasses a total of 53 reproducible networks, categorized into seven domains based on their anatomical and functional attributes. These domains include subcortical, auditory, sensorimotor, visual, cognitive control, default mode, and cerebellar domains (Du et al. 2020). The experimental outcomes from the asymmetric fusion approach, involving spatial maps illustrating the links between multivariate ${\text{FCPs}}_{\text{asym}}$ and voxel‐wise GMV data, are presented in Figure 2. In this figure, five ${\text{FCPs}}_{\text{asym}}$ components are plotted along with the spatial maps showcasing the voxel‐wise correlations between GMV data and ${\mathrm{FCP}}_{\text{asym}}$ loadings. Our symmetric fusion approach results, depicted in Figure 3, exhibit spatial maps illustrating the connections between multivariate ${\text{FCPs}}_{\mathrm{sym}}$ and ${\text{SCPs}}_{\mathrm{sym}}$. This figure showcases five ${\mathrm{FCP}}_{\mathrm{sym}}$ components and their corresponding spatial maps of ${\mathrm{SCP}}_{\mathrm{sym}}$ components. Furthermore, the associations of ${\text{FCPs}}_{\mathrm{sym}}$ and ${\text{SCPs}}_{\mathrm{sym}}$ components with age are depicted using upper and lower arrows along with their associated *T*‐values. A high negative (or positive) *T*‐value signifies an increase (or decrease) in the expression of the specific change pattern with age (Saha et al. 2022), where the upper and lower arrows represent increasing and decreasing pattern changes with age, respectively.

The results from the asymmetric fusion approach reveal noteworthy modularity in the fMRI result, suggesting structured changes occurring over the 2‐year period. And, interestingly, these were linked to strong and unique patterns of structural changes. In Figure 2, significant connections between ${\text{FCPs}}_{\text{asym}}$ and voxel‐wise GMV data are evident. We also explored the covariation between the loading parameters. Notably, the correlation between loadings 4 and 2 of ${\text{FCPs}}_{\text{asym}}$ is −0.38. In the spatial map figure, Component 2 displays a significant positive correlation (FDR‐corrected, *p* = 2.45x‐06), while the subcortical domain (SC) shows a widespread negative correlation with altered GMV data. The voxel‐wise correlation map suggests that Components 2 and 4 of ${\text{FCPs}}_{\text{asym}}$ exhibit the same correlation with altered GMV data, but in opposite directions. Furthermore, a strong positive correlation (*r* = 0.35) between loadings 4 and 5 of ${\text{FCPs}}_{\text{asym}}$ is observed. Similar to Component 4, our voxel‐wise correlation map reveals a significant negative correlation between ${\text{FCPs}}_{\text{asym}}$ and voxel‐wise changed GMV data for Component 5 (FDR‐corrected, *p* = 9.83x‐04). Conversely, the SC shows a significant positive correlation, with notable differences in the subcortical region of the voxel‐wise correlation between Components 2 and 4. Component 4 demonstrates a higher voxel‐wise correlation in the SC.

The results from the symmetric fusion approach indicate that ${\text{FCPs}}_{\mathrm{sym}}$ associated with Components 2 and 3 exhibit significant changes with increasing age in the developing brain. Both components demonstrate an increasing trend with age, as evidenced by their negative *T*‐values. Component 3 shows increased brain functional connectivity between the visual (VS) and sensorimotor (SM) domains in the FNC data. Concurrently, there are decreasing changes in the bilateral sensorimotor cortex in the sMRI data over the 2‐year period. Moreover, the ${\mathrm{FCP}}_{\mathrm{sym}}$ of Component 3 exhibits a decreasing trend with age in functional connectivity between the VS and cerebellar (CB) domains, as well as between the SM and cognitive control (CC) domains. Furthermore, a two‐sample *t*‐test based on sex (biological sex assigned at birth) differences using the loading parameters from both modalities indicates that males show a smaller expression of change patterns in ${\mathrm{SCP}}_{\mathrm{sym}}$ for Component 2 compared to females.

### 3.2 Evaluation of Gender Showing Change Patterns Coupling

**FIGURE 4:**
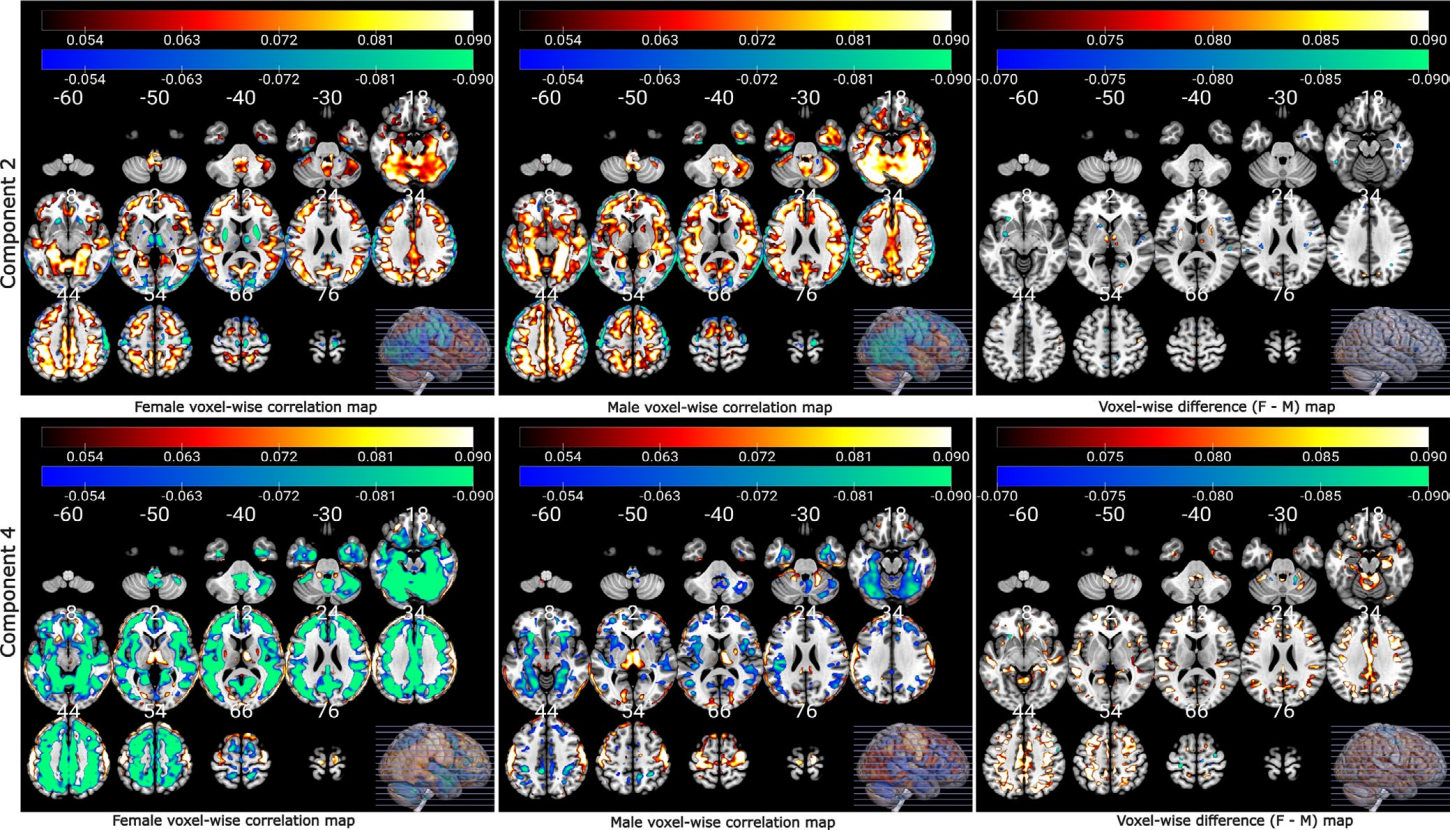
Voxel‐wise correlations of females, males, and distinctions in voxel‐wise correlations map (female—male) between male and female loading parameters for ${\text{FCPs}}_{\text{asym}}$ and raw ΔGMV. Females exhibit a stronger positive coupling for the ${\mathrm{FCP}}_{\text{asym}}$ Component 2 and stronger negative coupling for the ${\mathrm{FCP}}_{\text{asym}}$ Component 4 compared to males.

In the asymmetric multimodal fusion method, we have computed the difference (female–male) in the voxel‐wise correlation map between females and males to assess the gender effect. We found a positive correlation between the loading parameter of Component 2 and raw ΔGMV for both male and female data. Component 4 shows negative association with ΔGMV and significant correlation difference between males and females where females show higher voxel‐wise correlation than males as displayed in Figure 4. We conducted FDR correction on the correlation values with a significance threshold set at 0.001.

**FIGURE 5:**
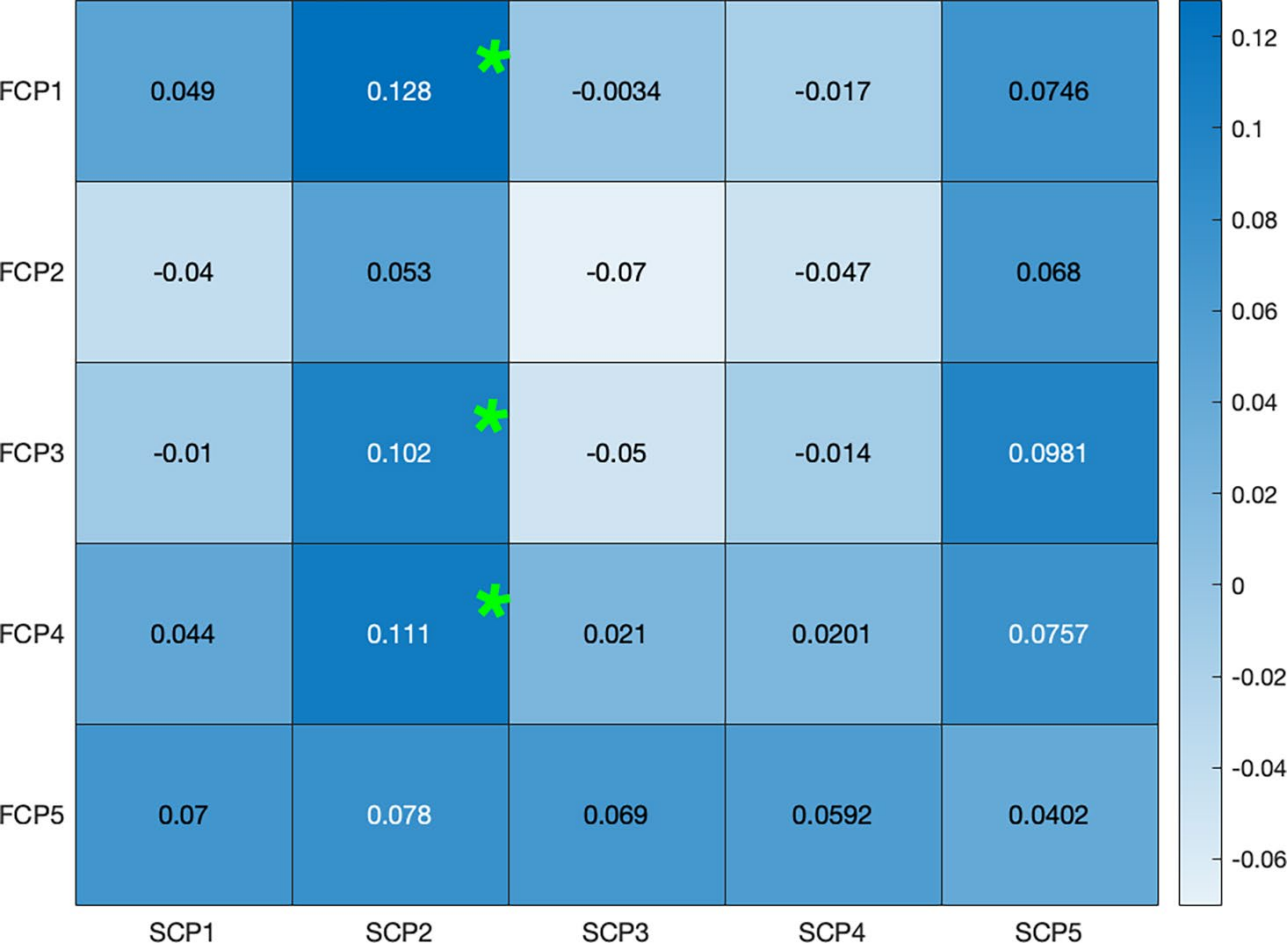
Differences in correlations between male and female loading parameters for ${\text{FCPs}}_{\mathrm{sym}}$ and ${\text{SCPs}}_{\mathrm{sym}}$. Females exhibit a stronger coupling between the ${\mathrm{FCP}}_{\mathrm{sym}}$ components 1, 3, and 4 and ${\mathrm{SCP}}_{\mathrm{sym}}$ Component 2 (indicated by *) expressions compared to males.

After applying the symmetric multimodal fusion technique, we computed the Pearson correlation between the loading parameters for all ${\text{FCPs}}_{\mathrm{sym}}$ and ${\text{SCPs}}_{\mathrm{sym}}$, separately for males and females. This analysis aimed to explore gender differences in coupling, specifically the associations at the subject expression level. The variations in correlations between loading parameters of ${\text{FCPs}}_{\mathrm{sym}}$ and ${\text{SCPs}}_{\mathrm{sym}}$, for males and females are visualized in Figure 5. Our findings indicate that females exhibited stronger coupling between ${\mathrm{SCP}}_{\mathrm{sym}}$, component 2 and ${\mathrm{FCP}}_{\mathrm{sym}}$ component 1 (Δ *r* = 0.128, FDR‐corrected, *p* = 2.1895e‐11), ${\mathrm{FCP}}_{\mathrm{sym}}$ component 3 (Δ *r* = 0.102, FDR‐corrected, *p* = 1.0081e‐07), and ${\mathrm{FCP}}_{\mathrm{sym}}$ component 4 (Δ *r* = 0.111, FDR‐corrected, *p* = 6.7136e‐09) compared to males.

### 3.3 Association of Changes in Multivariate Pattern Coupling With Psychopathology Score, and Cognitive Score

**FIGURE 6:**
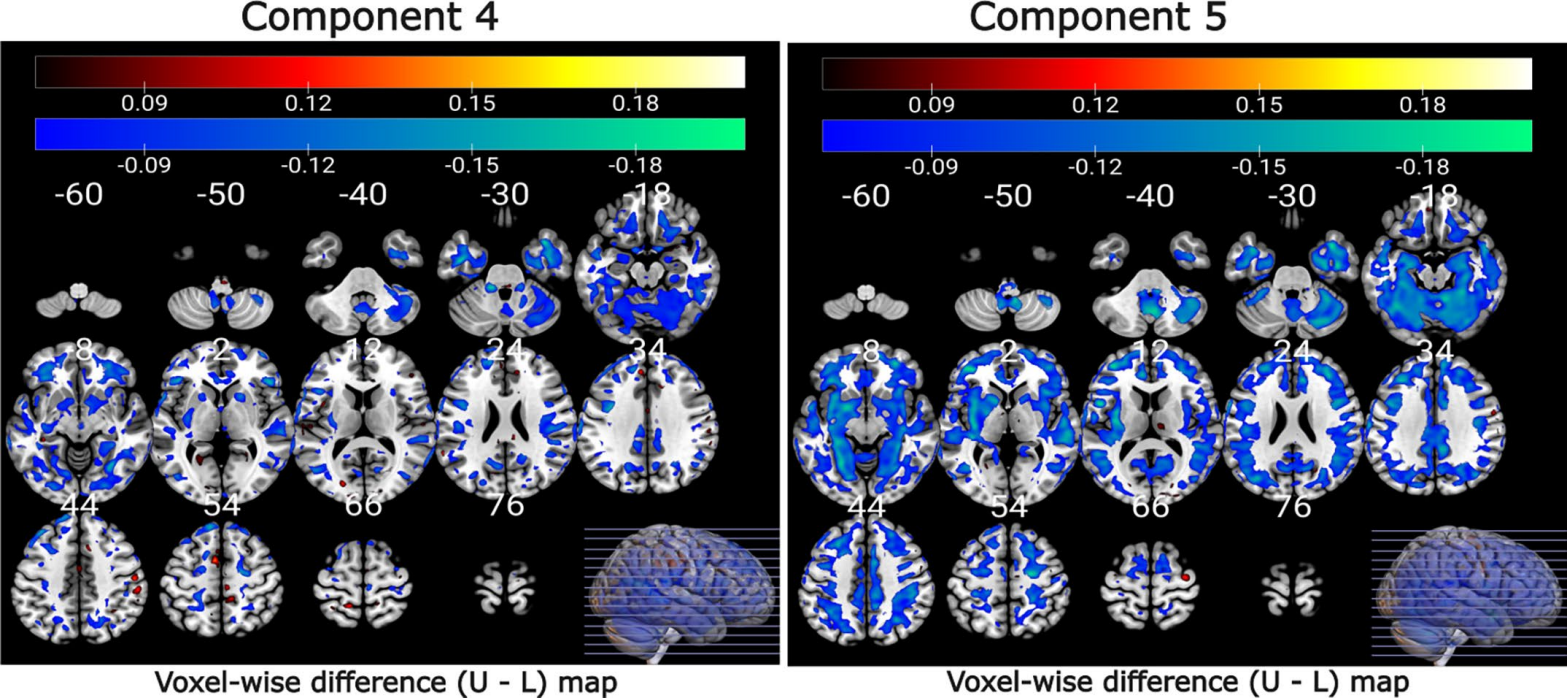
Voxel‐wise correlation difference map (upper–lower) illustrating the relationship between loading parameters of ${\text{FCPs}}_{\text{asym}}$, and raw ΔGMV for the upper and lower quartile of psychiatric scores. Components 4 and 5 exhibit notably stronger voxel‐wise correlations within the lower quartile of psychiatric scores (differences are negative).

**FIGURE 7:**
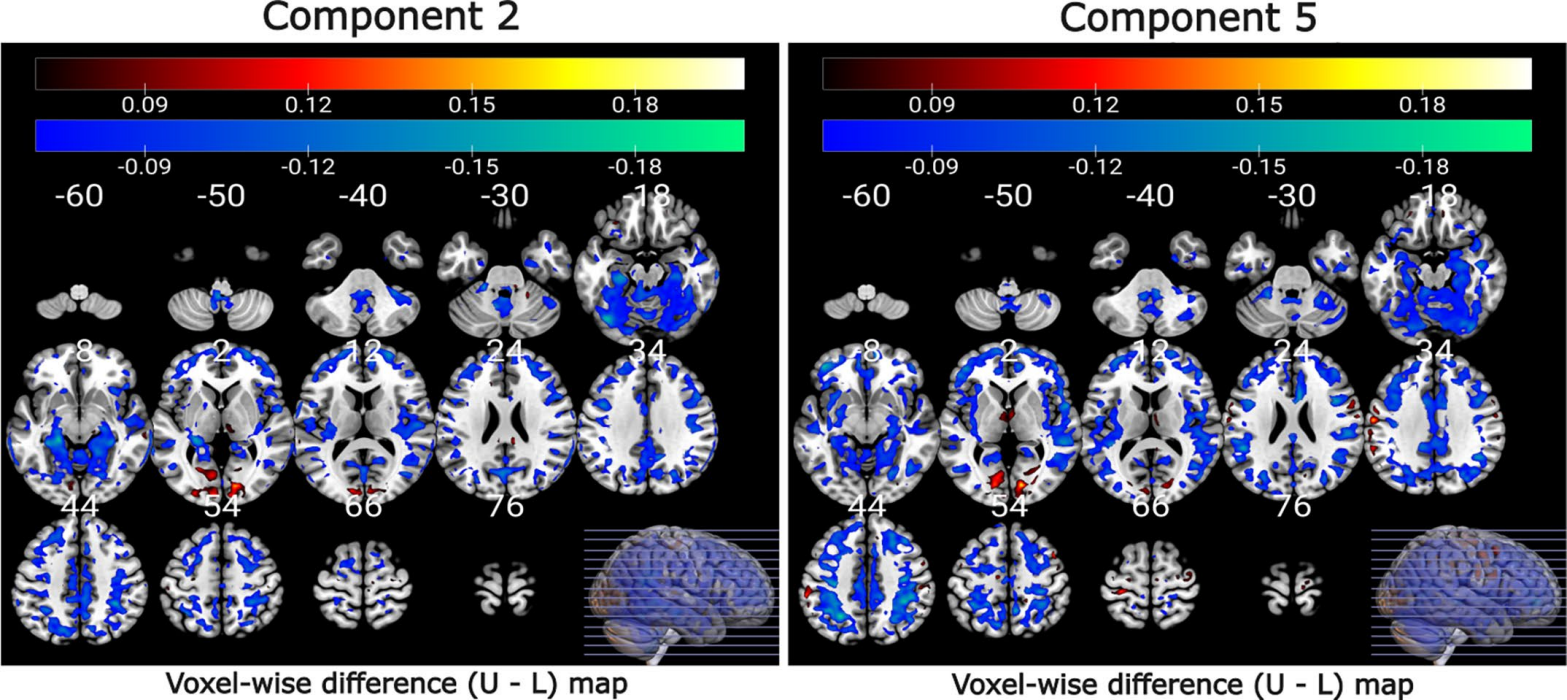
Voxel‐wise correlation difference map (upper–lower) illustrating the relationship between loading parameters of ${\text{FCPs}}_{\text{asym}}$ and raw ΔGMV for the upper and lower quartile of cognitive scores. Components 2 and 5 exhibit notably stronger voxel‐wise correlations within the lower quartile of cognitive scores.

We explored the relationship between changes in multivariate pattern coupling and subject measures, specifically psychopathology scores difference and cognitive scores difference. Our results reveal that both Components 4 and 5 exhibit a significant negative association with raw ΔGMV data within the lower quartile of psychiatric scores, as illustrated in Figure 6. The voxel‐wise correlation maps for cognitive scores are presented in Figure 7. Component 2 of ${\mathrm{FCP}}_{\text{asym}}$, predominantly shows a positive correlation with the ΔGMV data within the lower quartile of cognitive scores. Regarding Component 5, it displays negative correlations in the lower quartiles. We observed a noteworthy difference between the upper and lower quartiles for both psychopathology and cognitive scores. Furthermore, we applied FDR correction at a significance level of 0.05 to the correlation values and only significant results are shown in the figures.

## 4 Discussion

To the best of our knowledge, this is the first study to estimate the multivariate pattern coupling/covariations in structural and functional connectivity associated with age progression using a very large sample of nearly 2734 subjects in developing brain. Our study introduces two data‐driven multivariate multimodal fusion analyses to delineate change patterns in brain structure and function among healthy adolescents over a 2‐year period. The key findings include (1) identification of intriguing patterns in FNC and GMV data, demonstrating links to individual variations. Specifically, ${\mathrm{FCP}}_{\text{asym}}$ components 2 and 4 display significant associations between their loadings and voxel‐wise GMV data, (2) discovery of structured ${\text{FCPs}}_{\mathrm{sym}}$, such as enhanced connectivity between visual and sensorimotor domains or decreasing coupling trend between the SM and CC, correlated with ${\text{SCPs}}_{\mathrm{sym}}$ indicating alterations in the bilateral sensorimotor cortex, (3) observation of stronger coupling between brain functional and structural changes in females compared to males, unveiling gender‐related disparities, (4) recognition of multiple functional connectivity and gray matter volume data linked to longitudinal changes in psychopathology and cognition scores within the developing brain.

Understanding longitudinal changes in the developing brain is essential for comprehending cognitive development and neural maturation. Multimodal fusion of functional connectivity and sMRI provides a powerful approach to examining these complex developmental processes. This study highlights the intricate relationship between functional and structural changes over time. Using multimodal fusion, significant connections between ${\text{FCPs}}_{\text{asym}}$ and voxel‐wise GMV have been identified, supporting previous findings on multimodal imaging in brain maturation (Cao et al. 2017; Douaud et al. 2014). Asymmetric fusion reveals modularity trends over 2 years, suggesting structured developmental changes occurring during brain development (Smith et al. 2011). These findings further reinforce the interplay between functional and structural maturation, aligning with prior research linking functional connectivity to anatomical substrates (Damoiseaux and Greicius 2009; Honey et al. 2009; Vázquez‐Rodríguez et al. 2019). The study identified intriguing relationships, such as a− 0.38 correlation between loadings 4 and 2, revealing potential opposing effects in functional networks (Fjell et al. 2013). Components 2 and 4 show similar yet opposing correlations with GMV changes, highlighting region‐specific developmental patterns (Sheline et al. 2009). Spatial mapping underscores these variations, particularly in the SC domain, where contrasting correlations emphasize the significance of regional differences in brain maturation. These findings enrich our understanding of how specific brain regions follow distinct developmental trajectories, influencing cognitive and behavioral outcomes. These insights contribute to understanding how variations in brain development influence cognitive and behavioral outcomes.

The symmetric multimodal fusion approach, mCCA + jICA, reveals complex relationships between structural and functional changes over time. Previous studies (Alexander‐Bloch et al. 2013; Bassett et al. 2008; Hagmann et al. 2008; Honey et al. 2009; Michael et al. 2010; Olesen et al. 2003; Rykhlevskaia et al. 2008; Segall et al. 2012; Skudlarski et al. 2010; Van Den Heuvel et al. 2009; Yu et al. 2011) demonstrate clear associations between structural and functional connectivity, highlighting covarying patterns in longitudinal changes. These findings emphasize the interplay between structural alterations and functional connectivity in brain maturation, consistent with research on the mutual influence of structure and function during development (Douaud et al. 2014; Hermundstad et al. 2013). Our analysis shows that Component 2 and 3's ${\text{FCPs}}_{\mathrm{sym}}$ exhibit negative *T*‐values, indicating an age‐related increase in adolescents. Component 3 reveals enhanced functional connectivity between the VS and SM domains, critical for integrating visual and motor information to support perception, action, and cognition (Kaufmann et al. 2015). This evidence suggests that the developing brain forms connections among various regions while adapting to external stimuli, which likely play a critical role in distinguishing between regular and irregular neural dynamics. The enhanced connectivity between visual and sensorimotor domains aligns with prior research on their dynamic interconnectivity (Dayan and Cohen 2011; Ungerleider and Haxby 1994), highlighting the role of visual inputs in motor coordination and sensorimotor integration. We observed decreased functional coupling between the VS‐CB and SM‐CC regions with age, indicated by the highest positive *T*‐values, suggesting neural network reorganization. The correlation between ${\mathrm{FCP}}_{\mathrm{sym}}$ and ${\mathrm{SCP}}_{\mathrm{sym}}$ alterations, particularly in the bilateral sensorimotor cortex, underscores the link between functional connectivity and structural modifications. These findings reveal covarying patterns of structural and functional dynamics during development, emphasizing their close relationship (Hagmann et al. 2008). Studies also suggest that changes in neuronal functioning correspond to alterations in gray matter structure (Schultz et al. 2012). Overall, understanding how structural changes covary with functional connectivity offers critical insights into the coordinated and interdependent mechanisms driving brain maturation (Dennis et al. 2013; Supekar et al. 2010).

In this study, we employed both asymmetric and symmetric multimodal fusion methods to explore gender effects on functional and structural change coupling, aiming to discern nuanced differences in neural dynamics between males and females. In the asymmetric multimodal fusion method, the examination of voxel‐wise correlation maps revealed intriguing gender effects. Specifically, a positive correlation between the loading parameter of Component 2 and raw ΔGMV was observed for both males and females. This finding is in line with previous studies indicating gender‐related differences in brain structure and connectivity, suggesting that both gender exhibit similar patterns of association between specific components and structural changes (Ingalhalikar et al. 2014). However, Component 4 exhibited a gender‐specific association with ΔGMV, demonstrating a significant correlation difference between males and females. Females exhibited higher voxel‐wise correlation than males, suggesting a nuanced gender‐related divergence in the relationship between this specific ${\mathrm{FCP}}_{\text{asym}}$ and structural changes. Gender‐specific differences in brain structure and connectivity have been previously reported (Ruigrok et al. 2014), and the present study adds granularity by highlighting component‐specific distinctions. The application of the symmetric multimodal fusion technique further delved into gender differences in coupling at the subject expression level. The results revealed distinct variations in coupling patterns between males and females, shedding light on gender‐dependent associations within the neural network. Notably, females exhibited stronger coupling between ${\mathrm{SCP}}_{\mathrm{sym}}$ component 2 and ${\mathrm{FCP}}_{\mathrm{sym}}$ components 1, 3, and 4 compared to males. This discovery indicates that women who play a major role in the structural change pattern (${\mathrm{SCP}}_{\mathrm{sym}}$ component 2) also make substantial contributions to the ${\text{FCPs}}_{\mathrm{sym}}$ of Components 1, 3, and 4. These observations suggest gender‐dependent variations in the interplay between structural changes and functional connectivity, emphasizing the importance of considering individual differences in understanding brain dynamics (Ingalhalikar et al. 2014; Ritchie et al. 2018). Based on the correlation values we have, it can be inferred that females demonstrate a stronger association between the function and structure of the developing brain than males.

Our analysis uncovered several functional and structural connectivity change pattern coupling associated with longitudinal changes in psychopathology and cognition scores within the developing brain. Specifically, we observed a significantly higher negative correlation between ${\mathrm{FCP}}_{\text{asym}}$ components 4 and 5 and raw ΔGMV in subjects with low psychiatric scores compared to those with high psychiatric scores. This finding implies that individuals with lower psychiatric scores demonstrate a stronger association between ${\text{FCPs}}_{\text{asym}}$, such as increased brain functional coupling between the VS‐SM and CB‐SC, or a decrease in change patterns between VS‐CB and SM‐SC, and raw ΔGMV. Additionally, ${\mathrm{FCP}}_{\text{asym}}$ components 2 and 5 exhibit positive and negative associations, respectively, with the ΔGMV data within the lower quartile of cognitive scores. This indicates that individuals with lower cognitive scores show both positive (Component 2) and negative (Component 5) voxel‐wise correlations. Notably, a significant higher correlation is observed between both ${\mathrm{FCP}}_{\text{asym}}$ components 2 and 5 and raw ΔGMV within the lower quartile of cognitive scores compared to the upper quartile. This suggests that individuals contributing less to changes in cognitive scores from baseline to 2 years exhibit a stronger association between functional and structural data in the developing brain. These findings align with recent studies that have reported age‐related changes in vasculature, brain anatomy, and brain function collectively contributing to complex interactions that influence cognitive alterations (Fabiani et al. 2021; Zimmerman et al. 2021). We also analyzed the impact of site on our analysis by running the model with and without site nuisance regressors. The results showed no significant influence of site information on our findings.

In this manuscript, we present an innovative method that combines various data types to analyze patterns across multiple domains. We apply this method to explore the connection between brain structure and function using a combination of FNC‐sMRI data. Several promising research directions are apparent for further exploration. For instance, expanding the use of additional imaging techniques could enhance our understanding of overall brain change patterns, a path we aim to explore in future investigations. Furthermore, future research should delve into how increased component numbers affect these couplings, where varying the component count may reveal stronger connections between different brain regions. Our proposed methods also have certain constraints that warrant consideration. First, it is important to note that while mCCA + jICA operates on ICA components instead of the original imaging data (e.g., using 3D contrast images instead of 4D fMRI data), this approach may lose some temporal information. However, working with these ICA components presents advantages such as reduced dimensionality (Calhoun and Adali 2008) and a simplified space for linking the data (Smith et al. 2009). Second, our utilization of ICA assumes linearity in capturing brain functional pattern changes. Nonetheless, recent research by Motlaghian et al. highlights the potential presence of nonlinear relationships within functional networks, a factor often overlooked in linear analyses (Motlaghian et al. 2022). Exploring nonlinear methodologies could offer valuable insights into age‐related functional change patterns in the developing brain. Finally, this study utilized data from youths aged 9–10 years, potentially indicating relatively low levels of psychiatric symptomatology among the subjects (Escrichs et al. 2021). However, it is anticipated that their psychopathology load may increase during adolescence (Paus et al. 2008). This relationship between brain and behavior might evolve, growing stronger, weaker, or altering in some other way, which could be directly examined in future waves of longitudinal ABCD data. This would enable the formulation of clear hypotheses.

## 5 Conclusion

In this study, we propose asymmetric and symmetric multimodal fusion techniques to examine longitudinal brain changes in adolescents. These methods explore structural and functional changes and their correlations with aging using FNC matrices and GMV data from the ABCD dataset. Our asymmetric fusion results reveal significant associations between FCP components 2 and 4 and voxel‐wise GMV, suggesting that multivariate FNC and GMV patterns relate to aging. The symmetric fusion technique identifies structured ${\text{FCPs}}_{\mathrm{sym}}$, such as increased visual‐sensorimotor connectivity and decreased sensorimotor‐cognitive control connectivity, correlated with ${\text{SCPs}}_{\mathrm{sym}}$ in the bilateral sensorimotor cortex. Studying functional connectivity–GMV coupling over time helps reveal how the developing brain modulates its structural and functional relationships for efficient processing. Our findings highlight brain components contributing to gender‐based differences and reveal functional–structural couplings linked to psychopathology and cognition. To our knowledge, our proposed methodologies for investigating age‐related change patterns and coupling in the developing brain is the first approach to assess whole‐brain covarying functional and structural changes in longitudinal data.

## Author Contributions

The study was designed by R.S. and V.D.C. Data preprocessing was performed by Z.F. The experimental design was contributed by R.S. and V.D.C. R.S. conducted the analyses, performed the statistical analysis, and drafted the initial manuscript. The manuscript was edited by V.D.C., D.K.S., Z.F., M.D., and R.F.S. All authors participated in various stages of experimental analyses and approved the final version of the manuscript.

## Conflicts of Interest

The authors declare no conflicts of interest.

## Supporting information


Data S1.

## Data Availability

The data that support the findings of this study are available on request from the corresponding author. The data are not publicly available due to privacy or ethical restrictions.

## Endnotes

^1^

https://abcdstudy.org/.

^2^

https://nda.nih.gov/.

^3^

https://abcdstudy.org/scientists/protocols‐mri/.

^4^

http://trendscenter.org/software/gift.

^5^

http://trendscenter.org/data.

^6^
SPM12http://www.fil.ion.ucl.ac.uk/spm/.

## References

[hbm70241-bib-0001] Abrol, A. , B. Rashid , S. Rachakonda , E. Damaraju , and V. D. Calhoun . 2017. “Schizophrenia Shows Disrupted Links Between Brain Volume and Dynamic Functional Connectivity.” Frontiers in Neuroscience 11: 624. 29163021 10.3389/fnins.2017.00624 PMC5682010

[hbm70241-bib-0002] Alexander‐Bloch, A. , J. N. Giedd , and E. Bullmore . 2013. “Imaging Structural Co‐Variance Between Human Brain Regions.” Nature Reviews Neuroscience 14, no. 5: 322–336. 23531697 10.1038/nrn3465 PMC4043276

[hbm70241-bib-0003] Bassett, D. S. , E. Bullmore , B. A. Verchinski , V. S. Mattay , D. R. Weinberger , and A. Meyer‐Lindenberg . 2008. “Hierarchical Organization of Human Cortical Networks in Health and Schizophrenia.” Journal of Neuroscience 28, no. 37: 9239–9248. 18784304 10.1523/JNEUROSCI.1929-08.2008 PMC2878961

[hbm70241-bib-0004] Bell, A. J. , and T. J. Sejnowski . 1995. “An Information‐Maximization Approach to Blind Separation and Blind Deconvolution.” Neural Computation 7, no. 6: 1129–1159. 7584893 10.1162/neco.1995.7.6.1129

[hbm70241-bib-0005] Benjamini, Y. , and Y. Hochberg . 1995. “Controlling the False Discovery Rate: A Practical and Powerful Approach to Multiple Testing.” Journal of the Royal Statistical Society. Series B, Statistical Methodology 57, no. 1: 289–300.

[hbm70241-bib-0006] Calhoun, V. D. , and T. Adali . 2008. “Feature‐Based Fusion of Medical Imaging Data.” IEEE Transactions on Information Technology in Biomedicine 13, no. 5: 711–720. 19273016 10.1109/TITB.2008.923773 PMC2737598

[hbm70241-bib-0007] Calhoun, V. D. , T. Adali , N. Giuliani , J. Pekar , K. Kiehl , and G. Pearlson . 2006. “Method for Multimodal Analysis of Independent Source Differences in Schizophrenia: Combining Gray Matter Structural and Auditory Oddball Functional Data.” Human Brain Mapping 27, no. 1: 47–62. 16108017 10.1002/hbm.20166 PMC6871470

[hbm70241-bib-0008] Calhoun, V. D. , and J. Sui . 2016. “Multimodal Fusion of Brain Imaging Data: A Key to Finding the Missing Link (s) in Complex Mental Illness.” Biological Psychiatry: Cognitive Neuroscience and Neuroimaging 1, no. 3: 230–244. 27347565 10.1016/j.bpsc.2015.12.005 PMC4917230

[hbm70241-bib-0009] Camara, E. , A. Rodriguez‐Fornells , and T. F. Münte . 2010. “Microstructural Brain Differences Predict Functional Hemodynamic Responses in a Reward Processing Task.” Journal of Neuroscience 30, no. 34: 11398–11402. 20739561 10.1523/JNEUROSCI.0111-10.2010 PMC6633350

[hbm70241-bib-0010] Cao, M. , Y. He , Z. Dai , et al. 2017. “Early Development of Functional Network Segregation Revealed by Connectomic Analysis of the Preterm Human Brain.” Cerebral Cortex 27, no. 3: 1949–1963. 26941380 10.1093/cercor/bhw038 PMC6059235

[hbm70241-bib-0011] Chen, K. , E. M. Reiman , Z. Huan , et al. 2009. “Linking Functional and Structural Brain Images With Multivariate Network Analyses: A Novel Application of the Partial Least Square Method.” NeuroImage 47, no. 2: 602–610. 19393744 10.1016/j.neuroimage.2009.04.053 PMC2700206

[hbm70241-bib-0012] Correa, N. M. , T. Eichele , T. Adalı , Y.‐O. Li , and V. D. Calhoun . 2010. “Multi‐Set Canonical Correlation Analysis for the Fusion of Concurrent Single Trial Erp and Functional Mri.” NeuroImage 50, no. 4: 1438–1445. 20100584 10.1016/j.neuroimage.2010.01.062 PMC2857695

[hbm70241-bib-0013] Correa, N. M. , Y.‐O. Li , T. Adali , and V. D. Calhoun . 2009. “Fusion of Fmri, Smri, and Eeg Data Using Canonical Correlation Analysis.” In IEEE International Conference on Acoustics, Speech and Signal Processing, 385–388. IEEE, 2009.

[hbm70241-bib-0014] Damoiseaux, J. S. , and M. D. Greicius . 2009. “Greater Than the Sum of Its Parts: A Review of Studies Combining Structural Connectivity and Resting‐State Functional Connectivity.” Brain Structure and Function 213: 525–533. 19565262 10.1007/s00429-009-0208-6

[hbm70241-bib-0015] Dayan, E. , and L. G. Cohen . 2011. “Neuroplasticity Subserving Motor Skill Learning.” Neuron 72, no. 3: 443–454. 22078504 10.1016/j.neuron.2011.10.008 PMC3217208

[hbm70241-bib-0016] Dennis, E. L. , N. Jahanshad , K. L. McMahon , et al. 2013. “Development of Brain Structural Connectivity Between Ages 12 and 30: A 4‐Tesla Diffusion Imaging Study in 439 Adolescents and Adults.” NeuroImage 64: 671–684. 22982357 10.1016/j.neuroimage.2012.09.004 PMC3603574

[hbm70241-bib-0017] DeRamus, T. , A. Iraji , Z. Fu , et al. 2021. “Stability of Functional Network Connectivity (Fnc) Values Across Multiple Spatial Normalization Pipelines in Spatially Constrained Independent Component Analysis.” In 2021 IEEE 21st International Conference on Bioinformatics and Bioengineering (BIBE), 1–6. IEEE.

[hbm70241-bib-0018] Douaud, G. , A. R. Groves , C. K. Tamnes , et al. 2014. “A Common Brain Network Links Development, Aging, and Vulnerability to Disease.” Proceedings of the National Academy of Sciences 111, no. 49: 17648–17653. 10.1073/pnas.1410378111 PMC4267352 25422429

[hbm70241-bib-0019] Du, Y. , Z. Fu , J. Sui , et al. 2020. “Neuromark: An Automated and Adaptive Ica Based Pipeline to Identify Reproducible Fmri Markers of Brain Disorders.” NeuroImage: Clinical 28: 102375. 32961402 10.1016/j.nicl.2020.102375 PMC7509081

[hbm70241-bib-0020] Escrichs, A. , C. Biarnes , J. Garre‐Olmo , et al. 2021. “Whole‐Brain Dynamics in Aging: Disruptions in Functional Connectivity and the Role of the Rich Club.” Cerebral Cortex 31, no. 5: 2466–2481. 33350451 10.1093/cercor/bhaa367

[hbm70241-bib-0021] Fabiani, M. , B. Rypma , and G. Gratton . 2021. “Aging and Cerebrovascular Health: Structural, Functional, Cognitive, and Methodological Implications.” Psychophysiology 58, no. 7: e13842. 34021598 10.1111/psyp.13842 PMC8217301

[hbm70241-bib-0022] Fjell, A. M. , L. T. Westlye , H. Grydeland , et al. 2013. “Critical Ages in the Life Course of the Adult Brain: Nonlinear Subcortical Aging.” Neurobiology of Aging 34, no. 10: 2239–2247. 23643484 10.1016/j.neurobiolaging.2013.04.006 PMC3706494

[hbm70241-bib-0023] Fu, Z. , J. Liu , M. Salman , J. Sui , and V. Calhoun . “Functional Connectivity Uniqueness and Stability? a Signature of Cognitive and Psychiatric Problems in Children.” 10.1109/EMBC48229.2022.9871390 36085708

[hbm70241-bib-0024] Groves, A. R. , C. F. Beckmann , S. M. Smith , and M. W. Woolrich . 2011. “Linked Independent Component Analysis for Multimodal Data Fusion.” NeuroImage 54, no. 3: 2198–2217. 20932919 10.1016/j.neuroimage.2010.09.073

[hbm70241-bib-0025] Hagmann, P. , L. Cammoun , X. Gigandet , et al. 2008. “Mapping the Structural Core of Human Cerebral Cortex.” PLoS Biology 6, no. 7: e159. 18597554 10.1371/journal.pbio.0060159 PMC2443193

[hbm70241-bib-0026] Hermundstad, A. M. , D. S. Bassett , K. S. Brown , et al. 2013. “Structural Foundations of Resting‐State and Task‐Based Functional Connectivity in the Human Brain.” Proceedings of the National Academy of Sciences 110, no. 15: 6169–6174. 10.1073/pnas.1219562110 PMC3625268 23530246

[hbm70241-bib-0027] Honey, C. J. , O. Sporns , L. Cammoun , et al. 2009. “Predicting Human Resting‐State Functional Connectivity From Structural Connectivity.” Proceedings of the National Academy of Sciences 106, no. 6: 2035–2040. 10.1073/pnas.0811168106 PMC2634800 19188601

[hbm70241-bib-0028] Ingalhalikar, M. , A. Smith , D. Parker , et al. 2014. “Sex Differences in the Structural Connectome of the Human Brain.” Proceedings of the National Academy of Sciences 111, no. 2: 823–828. 10.1073/pnas.1316909110 PMC3896179 24297904

[hbm70241-bib-0029] Kaufmann, T. , K. C. Skåtun , D. Alnæs , et al. 2015. “Disintegration of Sensorimotor Brain Networks in Schizophrenia.” Schizophrenia Bulletin 41, no. 6: 1326–1335. 25943122 10.1093/schbul/sbv060 PMC4601711

[hbm70241-bib-0030] Kim, S.‐G. , W. H. Jung , S. N. Kim , J. H. Jang , and J. S. Kwon . 2015. “Alterations of Gray and White Matter Networks in Patients With Obsessive‐Compulsive Disorder: A Multimodal Fusion Analysis of Structural Mri and Dti Using Mcca+ Jica.” PLoS One 10, no. 6: e0127118. 26038825 10.1371/journal.pone.0127118 PMC4454537

[hbm70241-bib-0031] Kong, X. , and P. S. Yu . 2014. “Brain Network Analysis: A Data Mining Perspective.” ACM SIGKDD Explorations Newsletter 15, no. 2: 30–38.

[hbm70241-bib-0032] Lin, F.‐H. , A. R. McIntosh , J. A. Agnew , G. F. Eden , T. A. Zeffiro , and J. W. Belliveau . 2003. “Multivariate Analysis of Neuronal Interactions in the Generalized Partial Least Squares Framework: Simulations and Empirical Studies.” NeuroImage 20, no. 2: 625–642. 14568440 10.1016/S1053-8119(03)00333-1

[hbm70241-bib-0033] Mesulam, M. 2000. “Brain, Mind, and the Evolution of Connectivity.” Brain and Cognition 42, no. 1: 4–6. 10739582 10.1006/brcg.1999.1145

[hbm70241-bib-0034] Michael, A. M. , S. A. Baum , T. White , et al. 2010. “Does Function Follow Form?: Methods to Fuse Structural and Functional Brain Images Show Decreased Linkage in Schizophrenia.” NeuroImage 49, no. 3: 2626–2637. 19733247 10.1016/j.neuroimage.2009.08.056 PMC2911118

[hbm70241-bib-0035] Motlaghian, S. M. , A. Belger , J. R. Bustillo , et al. 2022. “Nonlinear Functional Network Connectivity in Resting Functional Magnetic Resonance Imaging Data.” Human Brain Mapping 43, no. 15: 4556–4566. 35762454 10.1002/hbm.25972 PMC9491296

[hbm70241-bib-0036] Ogawa, S. , T.‐M. Lee , A. R. Kay , and D. W. Tank . 1990. “Brain Magnetic Resonance Imaging With Contrast Dependent on Blood Oxygenation.” Proceedings of the National Academy of Sciences 87, no. 24: 9868–9872. 10.1073/pnas.87.24.9868 PMC55275 2124706

[hbm70241-bib-0037] Olesen, P. J. , Z. Nagy , H. Westerberg , and T. Klingberg . 2003. “Combined Analysis of Dti and Fmri Data Reveals a Joint Maturation of White and Grey Matter in a Fronto‐Parietal Network.” Cognitive Brain Research 18, no. 1: 48–57. 14659496 10.1016/j.cogbrainres.2003.09.003

[hbm70241-bib-0038] Ouyang, X. , K. Chen , L. Yao , et al. 2015. “Simultaneous Changes in Gray Matter Volume and White Matter Fractional Anisotropy in Alzheimer's Disease Revealed by Multimodal Cca and Joint Ica.” Neuroscience 301: 553–562. 26116521 10.1016/j.neuroscience.2015.06.031 PMC4522191

[hbm70241-bib-0039] Paus, T. , M. Keshavan , and J. N. Giedd . 2008. “Why Do Many Psychiatric Disorders Emerge During Adolescence?” Nature Reviews Neuroscience 9, no. 12: 947–957. 19002191 10.1038/nrn2513 PMC2762785

[hbm70241-bib-0040] Ritchie, S. J. , S. R. Cox , X. Shen , et al. 2018. “Sex Differences in the Adult Human Brain: Evidence From 5216 UK Biobank Participants.” Cerebral Cortex 28, no. 8: 2959–2975. 29771288 10.1093/cercor/bhy109 PMC6041980

[hbm70241-bib-0041] Ruigrok, A. N. , G. Salimi‐Khorshidi , M.‐C. Lai , et al. 2014. “A Meta‐Analysis of Sex Differences in Human Brain Structure.” Neuroscience and Biobehavioral Reviews 39: 34–50. 24374381 10.1016/j.neubiorev.2013.12.004 PMC3969295

[hbm70241-bib-0042] Rykhlevskaia, E. , G. Gratton , and M. Fabiani . 2008. “Combining Structural and Functional Neuroimaging Data for Studying Brain Connectivity: A Review.” Psychophysiology 45, no. 2: 173–187. 17995910 10.1111/j.1469-8986.2007.00621.x

[hbm70241-bib-0043] Saha, R. , D. K. Saha , M. A. Rahaman , Z. Fu , and V. D. Calhoun . 2022. “Longitudinal Whole‐Brain Functional Network Change Patterns Over a Two‐Year Period in the Abcd Data.” In IEEE 19th International Symposium on Biomedical Imaging (ISBI), 1–4. IEEE, 2022.

[hbm70241-bib-0044] Saha, R. , D. K. Saha , M. A. Rahaman , Z. Fu , J. Liu , and V. D. Calhoun . 2024. “A Method to Estimate Longitudinal Change Patterns in Functional Network Connectivity of the Developing Brain Relevant to Psychiatric Problems, Cognition, and Age.” Brain Connectivity 14, no. 2: 130–140. 38308475 10.1089/brain.2023.0040 PMC10954605

[hbm70241-bib-0045] Schultz, C. C. , P. Fusar‐Poli , G. Wagner , et al. 2012. “Multimodal Functional and Structural Imaging Investigations in Psychosis Research.” European Archives of Psychiatry and Clinical Neuroscience 262: 97–106. 10.1007/s00406-012-0360-5 22940744

[hbm70241-bib-0046] Segall, J. M. , E. A. Allen , R. E. Jung , et al. 2012. “Correspondence Between Structure and Function in the Human Brain at Rest.” Frontiers in Neuroinformatics 6: 10. 22470337 10.3389/fninf.2012.00010 PMC3313067

[hbm70241-bib-0047] Sheline, Y. I. , D. M. Barch , J. L. Price , et al. 2009. “The Default Mode Network and Self‐Referential Processes in Depression.” Proceedings of the National Academy of Sciences 106, no. 6: 1942–1947. 10.1073/pnas.0812686106 PMC2631078 19171889

[hbm70241-bib-0048] Skudlarski, P. , K. Jagannathan , K. Anderson , et al. 2010. “Brain Connectivity Is Not Only Lower but Different in Schizophrenia: A Combined Anatomical and Functional Approach.” Biological Psychiatry 68, no. 1: 61–69. 20497901 10.1016/j.biopsych.2010.03.035 PMC2900394

[hbm70241-bib-0049] Smith, S. M. , P. T. Fox , K. L. Miller , et al. 2009. “Correspondence of the Brain's Functional Architecture During Activation and Rest.” Proceedings of the National Academy of Sciences 106, no. 31: 13040–13045. 10.1073/pnas.0905267106 PMC2722273 19620724

[hbm70241-bib-0050] Smith, S. M. , K. L. Miller , G. Salimi‐Khorshidi , et al. 2011. “Network Modelling Methods for Fmri.” NeuroImage 54, no. 2: 875–891. 20817103 10.1016/j.neuroimage.2010.08.063

[hbm70241-bib-0051] Sui, J. , H. He , G. D. Pearlson , et al. 2013. “Three‐Way (n‐Way) Fusion of Brain Imaging Data Based on Mcca+ Jica and Its Application to Discriminating Schizophrenia.” NeuroImage 66: 119–132. 23108278 10.1016/j.neuroimage.2012.10.051 PMC3897558

[hbm70241-bib-0052] Sui, J. , R. Huster , Q. Yu , J. M. Segall , and V. D. Calhoun . 2014. “Function–Structure Associations of the Brain: Evidence From Multimodal Connectivity and Covariance Studies.” NeuroImage 102: 11–23. 24084066 10.1016/j.neuroimage.2013.09.044 PMC3969780

[hbm70241-bib-0053] Sui, J. , G. Pearlson , A. Caprihan , et al. 2011. “Discriminating Schizophrenia and Bipolar Disorder by Fusing Fmri and Dti in a Multimodal Cca+ Joint Ica Model.” NeuroImage 57, no. 3: 839–855. 21640835 10.1016/j.neuroimage.2011.05.055 PMC3129373

[hbm70241-bib-0054] Sui, J. , Q. Yu , H. He , G. D. Pearlson , and V. D. Calhoun . 2012. “A Selective Review of Multimodal Fusion Methods in Schizophrenia.” Frontiers in Human Neuroscience 6: 27. 22375114 10.3389/fnhum.2012.00027 PMC3285795

[hbm70241-bib-0055] Supekar, K. , L. Q. Uddin , K. Prater , H. Amin , M. D. Greicius , and V. Menon . 2010. “Development of Functional and Structural Connectivity Within the Default Mode Network in Young Children.” NeuroImage 52, no. 1: 290–301. 20385244 10.1016/j.neuroimage.2010.04.009 PMC2976600

[hbm70241-bib-0056] Ungerleider, L. G. , and J. V. Haxby . 1994. “What'and ‘Where’in the Human Brain.” Current Opinion in Neurobiology 4, no. 2: 157–165. 8038571 10.1016/0959-4388(94)90066-3

[hbm70241-bib-0057] Van Den Heuvel, M. P. , R. C. Mandl , R. S. Kahn , and H. E. Hulshoff Pol . 2009. “Functionally Linked Resting‐State Networks Reflect the Underlying Structural Connectivity Architecture of the Human Brain.” Human Brain Mapping 30, no. 10: 3127–3141. 19235882 10.1002/hbm.20737 PMC6870902

[hbm70241-bib-0058] Vázquez‐Rodríguez, B. , L. E. Suárez , R. D. Markello , et al. 2019. “Gradients of Structure–Function Tethering Across Neocortex.” Proceedings of the National Academy of Sciences 116, no. 42: 21219–21227. 10.1073/pnas.1903403116 PMC6800358 31570622

[hbm70241-bib-0059] Yu, Q. , J. Sui , S. Rachakonda , H. He , G. Pearlson , and V. D. Calhoun . 2011. “Altered Small‐World Brain Networks in Temporal Lobe in Patients With Schizophrenia Performing an Auditory Oddball Task.” Frontiers in Systems Neuroscience 5: 7. 21369355 10.3389/fnsys.2011.00007 PMC3037777

[hbm70241-bib-0060] Zhang, D. , Y. Wang , L. Zhou , et al. 2011. “Multimodal Classification of Alzheimer's Disease and Mild Cognitive Impairment.” NeuroImage 55, no. 3: 856–867. 21236349 10.1016/j.neuroimage.2011.01.008 PMC3057360

[hbm70241-bib-0061] Zimmerman, B. , B. Rypma , G. Gratton , and M. Fabiani . 2021. “Age‐Related Changes in Cerebrovascular Health and Their Effects on Neural Function and Cognition: A Comprehensive Review.” Psychophysiology 58, no. 7: e13796. 33728712 10.1111/psyp.13796 PMC8244108

