# Supplementary material for: Analysis of Longitudinal Change Patterns in Developing Brain Using Functional and Structural Magnetic Resonance Imaging via Multimodal Fusion
Source: Hum Brain Mapp. 2025 Jul 3;46(10):e70241. doi: 10.1002/hbm.70241 (PMC12223545; doi:10.1002/hbm.70241)
Supplement: Supplementary file 1 — Data S1. [file HBM-46-e70241-s001.pdf]

Supplementary Material

“Analysis of Longitudinal Change Patterns in Developing Brain Using Functional and Structural Magnetic Resonance Imaging via Multimodal Fusion”

Cognitive scores Variables

nihtbx_picvocab_fc
nihtbx_flanker_fc
nihtbx_pattern_fc
nihtbx_picture_fc
nihtbx_reading_fc
nihtbx_cryst_fc
nihtbx_picvocab_uncorrected
nihtbx_picvocab_agecorrected
nihtbx_flanker_uncorrected
nihtbx_flanker_agecorrected
nihtbx_pattern_uncorrected
nihtbx_pattern_agecorrected
nihtbx_picture_uncorrected
nihtbx_picture_agecorrected
nihtbx_reading_uncorrected
nihtbx_reading_agecorrected
nihtbx_cryst_uncorrected
nihtbx_cryst_agecorrected

Psychiatric problems scores Variables

cbcl_scr_07_sct_r	cbcl_scr_syn_internal_r
cbcl_scr_07_sct_t	cbcl_scr_syn_internal_t
cbcl_scr_07_ocd_r	cbcl_scr_syn_external_r
cbcl_scr_07_ocd_t	cbcl_scr_syn_external_t
cbcl_scr_07_stress_r	cbcl_scr_syn_somatic_r
cbcl_scr_07_stress_t	cbcl_scr_syn_somatic_t
cbcl_scr_dsm5_adhd_r	cbcl_scr_syn_social_r
cbcl_scr_dsm5_adhd_t	cbcl_scr_syn_social_t
cbcl_scr_dsm5_opposit_r	cbcl_scr_syn_thought_r
cbcl_scr_dsm5_opposit_t	cbcl_scr_syn_thought_t
cbcl_scr_dsm5_conduct_r	cbcl_scr_syn_attention_r
cbcl_scr_dsm5_conduct_t	cbcl_scr_syn_attention_t
cbcl_scr_syn_totprob_r	cbcl_scr_syn_rulebreak_r
cbcl_scr_syn_totprob_t	cbcl_scr_syn_rulebreak_t
cbcl_scr_dsm5_depress_r	cbcl_scr_syn_aggressive_r
cbcl_scr_dsm5_depress_t	cbcl_scr_syn_aggressive_t
cbcl_scr_dsm5_anxdisord_r	cbcl_scr_syn_anxdep_r
cbcl_scr_dsm5_anxdisord_t	cbcl_scr_syn_anxdep_t
cbcl_scr_dsm5_somaticpr_r	cbcl_scr_syn_withdep_r
cbcl_scr_dsm5_somaticpr_t	cbcl_scr_syn_withdep_t

We used the previously mentioned variables to create composite scores for cognitive and psychopathology assessments. Missing values in each variable were addressed by replacing them with the mean. Each variable was then standardized by subtracting its mean and dividing by its standard deviation. Finally, the scores were combined within two categories—cognition and psychiatric issues—to generate two separate composite variables.
